# COVID-19 Test Before Tokyo2020 Paralympic Games: An Implemented Protocol to Protect Paralympic Athletes

**DOI:** 10.3389/fspor.2022.834410

**Published:** 2022-02-16

**Authors:** Greta E. Muti, Giovanna Muti-Schuenemann, Fulvia Pimpinelli, Antonio Spataro, Antonio Fiore, Francesca Ciasullo, Daniela Olivieri, Marta Rigoni, Serena Delbue, Elena Pariani, Fabio Muzi, Sara Donzelli, Sabrina Strano, Aldo Morrone, Giovanni Blandino, Paola Muti

**Affiliations:** ^1^Medical Scholl, San Raffaele University, Milan, Italy; ^2^Health Research Methods, Evidence and Impact Department, McMaster University, Hamilton, ON, Canada; ^3^Lazio Region COVID Center, San Gallicano Institute, Instituti Fisioterapici Ospedalieri (IFO), Rome, Italy; ^4^Italian Rowing Federation Olympic Team, Comitato Olimpico Nazionale Italiano (CONI), Rome, Italy; ^5^Italian Fencing Federation Olympic Team, Comitato Olimpico Nazionale Italiano (CONI), Rome, Italy; ^6^Centro Italiano Studio Sviluppo Psicoterapie A Breve Termine (CISSPAT) Sport Psychology and Coaching Unit, Consultant Italian Fencing Olympic Federation, Rome, Italy; ^7^Consultant Italian Fencing Olympic Federation, Rome, Italy; ^8^Industrial Engineering Department, Trento University, Trento, Italy; ^9^Department of Biomedical, Surgical and Dental Sciences, University of Milan, Milan, Italy; ^10^Department of Biomedical Sciences for Health, University of Milan, Milan, Italy; ^11^Sport Medicine Department, Regional Health System, Terni, Italy; ^12^Oncogenomic and Epigenetic Unit, Italian National Cancer Center “Regina Elena”, Rome, Italy; ^13^Scientific Director Office, San Gallicano Institute, IFO, Rome, Italy

**Keywords:** COVID-19, Paralympic games, COVID-19 test, anti-COVID-19 protocol, COVID-19 pandemic

## Abstract

**Objectives:**

The COVID-19 pandemic represents a difficult challenge for the whole of humanity. Sports, in which contact between athletes is essential, became impossible to practice without the risk of viral spread. Athletes of the national teams are a particular subgroup of the population for whom there is an important need for protection and the implementation of targeted preventive measures. The present report describes the protocol that was developed to answer the urgent protection need for athletes during COVID-19 pandemic. The protocol aimed at demonstrating the feasibility of a rigid prevention intervention to prevent outbreaks and infections in terms of COVID-19 as well as in other potential future pandemics from pathogens with similar path of transmission.

**Methods:**

The study was conducted in rowing para-thletes training of the Paralympic Games in Tokyo2020. It was designed to create an anti-COVID-19 “*protection bubble*” with the aim to isolate para-athletes and their technical support team during pre-Olympic retreats. The “*bubble*” development relied on a carefully conducted protocol of repeated antigen and molecular COVID-19 tests on nasal and oropharyngeal fluids among all participants carried out before, during and at the end of each retreat.

**Results:**

During the 10 months of protocol implementation there were no COVID-19 outbreaks among the para-athletes and technical personnel during the retreats. In total, 552 PCR tests and 298 antigen-based tests were performed for an average number of 42 test per athlete. The number of retreat participants was larger (*n* = 23) in the beginning of the year due to the Paralympic selection rounds and smaller at the end of the study period (*n* = 12).

**Conclusion:**

The protocol has indicated that it is possible to implement an anti-COVID-19 protection protocol where athletes and technical staff can train and compete in safe conditions. The study showed that it is feasible to implement a rigid prevention protocol for athletes and technical staff based on repeated COVID-19 antigenic and molecular tests for a long period of training with excellent participation and compliance.

## Introduction

The SARS-CoV-2 pandemic has greatly modified and strained healthcare systems, disease outcomes and globalization. Since the World Health Organization (WHO) announced the outbreak as a Public Health Emergency of International Concern, at the end of January 2020, governments of multiple countries began implementing rigid protocols for the containment of the virus (WHO, 2020a). These protocols involved declaring a national state of emergency resulting in lockdowns, mask mandates and social distancing in every public place (Adhanom, 2020). As a result of the combined effect of lack of manpower and closing of non-essential businesses, the rates of unemployment and poverty greatly rose leading to a mental health crisis. Within the vulnerable populations suffering from these measures, professional athletes saw their daily life completely altered by the uncertainties of the pandemic and waiting for the confirmation of the Olympic and Paralympic Games.

Sports, in which contact between athletes is essential, became impossible to practice without the risk of viral spread. Therefore, to minimize the spread of SARS-CoV-2, multiple elite sporting events in 2020 and 2021 were either cancelled or postponed to a later date. By the end of March 2020 both the Olympic and Paralympic games, scheduled for July and August of the same year in Tokyo, were postponed to the following year (Kano, 2020). In addition to the anxiety related to the lack of information on the actual carrying out of the Olympic and Paralympic Games, professional athletes saw themselves greatly impacted by the lack of knowledge on how the SARS-Cov-2 infection could affect their physical and mental performance. Several studies have investigated the impact of SARS-CoV-2 on athletes' and parathletes' conditions, training activities, health and performance status (Hu et al., 2021; Shaw et al., 2021). A recent survey conducted in Polish Paralympic athletes at the beginning of the pandemic wave, found that they were strongly affected by both the pandemic and measures undertaken to slow its spread, especially by the lockdown (Urbański et al., 2021). Hu et al. (2021) have studied the effect of COVID19 pandemic on athletes' athletic identity, an important component of athlete's self-concept and health and performance outcomes. They found that, in particular Paralympic athletes felt that their athletic identity was negatively challenged by the SARS-COV-2 pandemic. This effect was explained by their singular focus on the loss of physical participation in sport during COVID-19. Kubosch et al. (2021) using a cross-sectional study design conducted as an online survey, found that due to the COVID-19 pandemic athletes were very worried about their health, their social contacts, and the social cohesion of our society even more than about their finances and sponsorships.

Follow-up studies on infected athletes often described constant cardiorespiratory residual alterations a long time after COVID-19 (Sala et al., 2020).

As an immediate consequence of these health concerns, a group of clinical researchers had urgently proposed a strict protocol of practical recommendations on how to exclude cardiorespiratory complications of COVID-19 in previously COVID-19 infected and returning-to-play elite athletes who placed high demand on their cardiorespiratory system (Hull et al., 2020; Wilson and Hull, 2020).

However, this strategy was not sufficient to protect the athletes from the COVID-19 health consequences because the risk of infection among them remained high. International and National Olympic and Paralympic Federations were looking for strategies to prevent SARS-Cov-2 infection through clear guidance and protocols based on a swab schedule or quarantine measures to control viral spread.

Here we describe the protocol that was developed and implemented to answer the urgent need of anti-pandemic protection for athletes. In particular, the protocol aimed at demonstrating the feasibility of a rigid prevention interventions to prevent outbreaks and infection during COVID-19 pandemic as well as in any other potential future pandemics based on similar infection transmission path. The protocol was based on a large collaboration across different Universities, Research Centers, Italian National Rowing Federation as well as across different competences such as epidemiology, microbiology, sport and exercise medicine and public health.

The protocol aimed also at providing National and International Sporting Federations with guidance and practical recommendations to allow athletes to train and perform under the safest conditions possible while preventing potential outbreaks amongst this community.

## Ethical Consideration

The protocol implementation was entirely supported by public funds made available by the anti-COVID19 Center of the Lazio Region, Italy. The protocol was approved by the University of Milan Institutional Review Board (Approval Number 02/21) and the informed consent was signed by all participants at the beginning of the study.

The protocol included only para-athletes (athletes that practice a sport where their physical impairments have an impact on their performance) and their support team staff.

## Study Design and Methods

### The Anti-COVID-19 Protection Bubble

The study was organized using an anti-COVID-19 protection bubble which had the aim to isolate para-athletes and technical support team during training in preparation of the Tokyo2020 Games. The “*bubble*” development was based on a carefully conducted protocol of repeated antigen and molecular COVID-19 tests on nasal and oropharyngeal fluids among all participants at the Paralympic preparation sessions carried out before, during and at the end of each retreat.

The “*bubble*” was created to offer para-athletes and the support personnel the opportunity to work, train and socialize in a special relax-no-fear-of-contamination environment. The “*bubble*” defined a completely closed environment where no one was allowed to enter without relevant health/technical reasons. As a matter of fact, if additional personnel (technical operators, physicians, physiotherapists) were needed, the protocol called for the additional personnel to perform a molecular test before entering in contact with para-athletes, coaches, and technicians already isolated. Their access was granted only with a “negative” result of the test.

The “bubble” was created for each retreat of the Italian Paralympic rowing team and in occasion of National and International rowing competitions.

The main outcome of this study was the evaluation of its feasibility. To evaluate the feasibility, we used the following criteria:

1) the prevalence of participation of Paralympic athletes / athletes and technical staff for the entire duration of the study (10 months);

2) the ability to collect nasal and oropharyngeal samples for the entire duration of the study by an athlete of the retreat team. This strategy was important to warrant both sample collection with standardized methods and regularity of their execution during retreats at the Olympic Center as well as at the different locations established for National and International rowing competitions;

3) the ability of the study field organization to transport regularly and on time the collected samples from the training and competition locations to the laboratories for the entire duration of the study;

4) the ability of the laboratories involved in the study to provide test results on time at the beginning, during and at the end of the training retreats for the entire duration of the study.

*Pararowing Retreats*: The Italian National Rowing Federation set a dedicated environment for all Pararowing retreats at the Center for preparation of the Olympic and Paralympic National Italian Rowing team in Piediluco in Umbria, Italy. The Olympic-Paralympic Center is in Piediluco directly overlooking the lake and it is a facility dedicated to the hospitality, preparation and training of athletes and para-athletes, technicians, and managers of Italian and International Rowing Federations. Together with state-of-the-art sports facilities and equipment, the Center is equipped with laboratories for biomechanical and physiological analysis, as well as facilities for the rehabilitation and recovery of injured athletes and para-athletes.

The samples for the protocol were collected at the Center in a dedicated health facility.

The “bubble” activation lasted for 10 months and corresponded to the Pre-Paralympic Games training period, from November 2020 to August 2021, ending during the last retreat before the departure for the Tokyo2020 Paralympic Games. Each retreat lasted from 7 to 14 days for a total of 11 retreats. The “bubble” remained active even after the athletes and technical staff vaccination received COVID-19 vaccination in June 2021.

### Competitions

There have been two international competitions during the protocol implementation period: the European Rowing Championships (Varese, Italy, April 6–11, 2021) and the International Pararowing Regatta—Paralympic Qualification Races (Gavirate, Italy, June 2–6, 2021). The “bubble” for competitions was developed when para-athletes arrived at the racing grounds and ended at the time of their departure. During the competitions para-athletes were asked to minimize contact with other national and international members not adhering to the same rigorous protocol, including teammates from the Italian Olympic rowing team as well as from other nations. During their stay all athletes were provided with the necessary equipment and information to prevent infection.

### Protocol Participants

For para-athletes practicing sport, competing, and socializing with team members and opponents is important from a rehabilitative perspective. Taking part in sports results in an increase in self-esteem and psychophysical resilience, which has also an important impact on the quality of life of this population (Urbański et al., 2021). The protocol focused on para-athletes because the risk of infection from SARS-Cov-2 is particularly high in these groups of athletes with visual and motor limitations due to the difficulty in maintaining the necessary precautions to avoid infection. For example, in some cases the inability to both wear facemasks and maintain the required safety distance increased the risk of being infected. These factors made the need of a COVID-19 prevention strategy particularly relevant for Paralympic athletes and this was the rational for their inclusion in the present protocol.

Participants in the retreats and competitions included males and females from the age of 18–55 years coming from different Italian regions. Athletes were divided into four categories based on Pararowing (PR) classifications (Table 1). The first category are the Coxswains, which are athletes that have no physical impairment and serve as guides to the visually impaired athletes. The PR3 category is dedicated to those athletes that have a permanent disability with remaining functional use of the legs, trunks and arms allowing them to slide the seat to propel the boat. This category is further subdivided into PR3 with visual impairment (PR3-VI) and PR3 with a physical disability (PR3-PD). Next, there is the PR2 category that includes athletes that have full functional use of only trunk and arms with weakened function or mobility in the legs that does not allow them to use the sliding seat in the boat. Lastly, there is the PR1 category composed of those athletes that have minimal or no trunk function and that strictly use their arms to propel the boat forward (Classifiers, 2017). The PR1 category is mostly composed of athletes with spinal cord injuries and that are wheelchair bound.

**Table 1:** Descriptive data of protocol participants and their residential region in Italy.

**Athlete**	**Gender**	**Age**	**MS**	**Region**	**CPR**	**PP**	**IBS**	**PR**
1	M	55	R	Lombardia	PR2	No	+	–
2	M	47	R	Lazio	PR1	No	–	–
3	F	42	S	Piemonte	PR3-VI	Yes	–	–
4	M	53	S	Liguria	PR2	Yes	–	–
5	F	46	S	Veneto	PR2	Yes	–	–
6	F	41	R	Piemonte	PR1	No	–	–
7	F	37	S	Lombardia	Coxwain	Yes	+	–
8	M	25	R	Campania	PR3-PD	Yes	–	–
9	M	54	R	Lombardia	PR1	No	+	+
10	M	25	R	Lazio	PR1	No	–	–
11	M	24	R	Piemonte	PR3-VI	Yes	+	–
12	F	25	S	Lazio	PR3-PD	No	+	+
13	F	27	S	Lombardia-Toscana	PR3-PD	Yes	–	–
14	F	26	S	Piemonte-Veneto	PR1	No	+	–
15	F	21	S	Campania	Coxwain	No	–	–
16	F	18	S	Lombardia	PR3-VI	No	+	–
Coaches								
1	M	55	S	Campania	–	Yes	–	–
2	M	64	R	Lombardia	–	Yes	+	–
3	F	25	S	Lombardia	–	No	+	–
4	M	25	S	Piemonte	–	No	–	–
5	M	55	R	Umbria	–	No	–	–
Manager								
1	M	62	R	Umbria	–	No	+	–
2	F	49	R	Umbria	–	Yes	–	–

*M, Male; F, Female; MS, Marital Status; R, Relationship; S, Single; CPR, Classification Pararowing; PR, Pararowing; PP, Paralympic Participants; IBS, Infection status before study; PR, Positive result at Retreat*.

The other participants included in the protocol were coaches, managers and technical staff that are there for support and training of the athletes.

The total number of participants was 23 (16 athletes, 5 coaches, and 2 staff), however the number of participants at each retreat changed because different coaches were requested to join retreats and different para-athletes were called or sorted throughout the selection process for the Paralympic Games. The final Paralympic rowing team was composed of 7 athletes, 3 coaches and 2 team managers.

## Material

### Antigen Test

SARS-Cov-2 antigen test was done at the arrival of the para-athletes and technical personnel, and it was immediately followed by the molecular test. The antigen test was implemented to immediately identify and isolate potential “positive” para-athletes coming from home while waiting for the result validation done by the PCR based test.

The NADAL^®^ COVID-19 Ag test was used as antigen test. This test involves lateral flow chromatographic immunoassay for the qualitative detection of SARS-CoV-2 viral nucleoprotein antigens in human nasopharyngeal or oropharyngeal samples.

### Molecular Test

At the COVID 19 Center of the Lazio Region, the molecular test was performed after RNA extraction by using Bosphore EX-Tract Dry Swab RNA Solution (AnatoliaGeneWork) according to manufacturer's instructions. Samples were tested for the presence of SARS-CoV-2 by the Bosphore Novel Coronavirus (2019-nCoV) v3 Gene Detection Kit based on the real-time Polymerase Chain Reaction (PCR).

At the COVID 19 Laboratory at the Milan University, the molecular test was performed after RNA extraction by QIAmp Viral RNA Mini Kit (QIAGEN), samples were tested for the presence of SARS-CoV-2 by specific one-step real-time RT-PCR assay according to WHO international guidelines and CDC protocol (Centers for Disease Control and Prevention (CDC), 2020).

The protocol required all PCR tests and antigen-based tests to always be executed by the one of the para-athletes. A medical student (GM) did all collections and performed all antigenic tests with two exceptions due to competing commitments. In these two cases the sport-medical doctor associated to that retreat period was asked to do the swabs and to follow the protocol procedure (FM co-author of the present report). GM and the sport-medical doctors who performed the test were trained to follow a standardized protocol for sample collection by FP at the COVID-19 Center of the Lazio Region (co-author of the present report). GM is also first author of the present report.

While the antigen tests were read by GM few minutes after their performance, the PCR-tests were transported by specifically requested independent service to the COVID-19 Center of the Lazio Region in Rome, where the swabs were analyzed by FP's team and the results communicated directly to the Italian Rowing Federation sport-medical doctor, the general coordinator of the protocol, the athlete-head coach and team coordinator.

During the two International Rowing competitions, the molecular tests were performed by the COVID19 Laboratory of the University of Milan under the supervision of SD and EP (co-authors of the present report) and transported there from the competition locations by voluntary service.

### Beginning of Retreat

Thus, at the beginning of each retreat oral-pharyngeal swabs were conducted on all athletes, technical and support staff participating in the retreat. As mentioned, the results of the PCR test would have been ready 5–6 h after biological sample collection, thus a provisional antigen-based test was done to allow athletes with negative antigen-based test to enter a pre-bubble environment. Even with a negative antigen-based test result, all participants were asked to always wear a mask and ensure social distancing in all the spaces at the retreat center and at the hotel. The retreat would only officially begin upon the arrival of all negative diagnostic test results.

Furthermore, by protocol, any subject negative at the antigen test but positive at PCR would have been immediately isolated and the original swab would be rerun for further validation by the laboratory in Rome. However, this last event, never occurred.

If any participant arrived after the creation of the “bubble,” they were asked to take a PCR test with a negative result at home 48 h before arriving and would undergo an antigen-based test upon arrival at the retreat.

### During Retreat

Once the initial diagnostic tests were done and the results had arrived, the bubble was created, and the retreat could begin. Because of the National Olympic-Medical Facility was in Rome at 100 km from the retreat center, it was necessary for para-athletes and technical personnel requiring medical assistance or follow-up visits to exit the bubble during the retreat to travel to Rome. These participants were requested to undergo an antigen-based test upon arrival to enter the bubble once more.

Furthermore, all retreat members were provided with all material and information regarding protection from infection. These included hotel staff, journalists, restaurant staff, medical staff, and other members of the Italian rowing federation.

Although the “bubble” allowed para-athletes to train without masks, social distancing was always maintained when possible. Sanitization of technical equipment was done before and after every training session. All training areas were only accessible to members of the retreat and a selected number of members of the rowing federation and medical staff. During the retreat, each and every single activity linked to training, consumption of meals and meetings took place in environments dedicated only to the participants of the “bubble” and therefore isolated from the other spaces. In particular, meals were given in a dedicated space of the hotel in which only “bubble” participants and negatively tested staff were allowed. The same dedicated area was also used for social activity and any other needs that athletes and coaches might have such as studying, one-on-one meetings, reading, etc. Other activities that were connected to training, and team meetings were always held at the Center for Olympic and Paralympic preparation. Every day the entire Center building was sanitized. All personnel that entered the Center were required to always follow the protocol which called for a PCR test, with a negative result before entering the Center, always followed by social distancing and mask use rules and to minimize contact with athletes and technical coaches as much as possible.

By protocol, all retreat members were asked to inform medical staff instantly if they developed COVID-19 symptoms. The most common COVID-19 symptoms on which the athletes were informed and alerted about were fever, cough, tiredness and loss of taste or smell, while less common symptoms were sore throat, headaches, pains, diarrhea, skin rashes and red irritated eyes (WHO, 2020b). It was always important to keep in mind when evaluating these symptoms that some of them could physiologically appear due to the high strenuous exercise performed on a daily basis. However, if there was a suspicion of infection an immediate isolation would be required, and antigen-based test would be performed. The antigen-based test was chosen for immediate action since it was always readily available, and the result would be given directly. In case the athlete resulted positive, the protocol called for all athletes and personnel that met the COVID-19 infected para-athlete to isolate immediately and be tested as well. All potential antigen-tested positive participants were then re-tested for validation by PCR test to activate the quarantine for those who were in contact with the positive case and involved in the retreat. Finally, all areas exposed to the “positive” individual would undergo a deep sanitization.

### End of Retreat

At the end of each retreat, a PCR test was done the evening before the participants' departure. This allowed for the results to arrive before departure and therefore prevent any further spread of SARS-CoV-2 upon arrival to their home regions. All members that resulted negative were allowed to go back home. In the case of a positive PCR test result the protocol required that the participant be put into immediate isolation and the original swab would be analyzed again to confirm positivity. As in the previous case, PCR validation was necessary to confirm the positive result. Furthermore, the protocol called for all athletes and personnel that met the COVID-19 infected person to be immediately isolated, immediately tested by PCR test and activate quarantine as in the case before.

## Results

The first source of infection for the team was due to potentially infected-yet-asymptomatic athletes who travelled from different regions in Italy characterized by different COVID-19 incidence and prevalence rates (Table 2). During this 10-month protocol implementation there were no COVID-19 outbreaks among the para-athletes and technical personnel during the retreats. In total 552 PCR tests (run at the beginning and at the end of each retreat) and 298 antigen-based tests were performed. The number of retreat participants was larger (*n* = 23) in the beginning of the year due to the Paralympic selection rounds taking place at the National Olympic and Paralympic Rowing Center in Piediluco (Table 1). After the selection process had taken place, the number of recruited athletes was smaller (*N* = 12) and remained constant until the departure for the Paralympic Games when it was further reduced to 7 athletes. A small variation in the number of tests conducted was due to additional Federal coaches invited to retreats to assist the para-rowing team performance.

**Table 2:** Date of retreats and number of participants per retreat.

**Dates**	**Type of retreat**	**Female participants**	**Male participants**	**Total participants**
18/11/20–22/11/20	Evaluating retreat	9	7	16
30/11/20–06/12/20	Evaluating retreat	9	8	17
15/12/20–21/12/20	Evaluating retreat	9	8	17
15/01/21–24/01/21	Evaluating retreat	10	10	20
12/02/21–22/02/21	Evaluating retreat	10	10	20
11/03/21–20/03/21	Evaluating retreat	9	10	19
28/03/21–02/04/21	Evaluating retreat	8	9	17
06/04/21–11/04/21	European championships	7	7	14
22/04/21–02/05/21	Evaluating retreat	8	10	18
09/05/21–14/05/21	Evaluating retreat	8	9	17
28/05/21–06/06/21	International pararowing regata	7	7	14
20/06/21–04/07/21	Evaluating retreat	6	6	12
14/07/21–29/07/21	Evaluating retreat	6	7	13
04/08/21–15/08/21	Evaluating retreat	6	6	12
18/08/21–02/09/21	Paralympics	5	6	11

In total, from the beginning of the pandemic, 7 athletes and 3 coaches were infected. Three athletes and two coaches resulted positive during lockdown period and before the beginning of the study. Other two athletes and one coach resulted positive after the European Championships in 2020 in Poznan before the beginning of the study. All the individuals that resulted positive were diagnosed through PCR tests performed by their local health centers.

During the study in two separate retreats, two athletes out of the seven who, at that point, entered the “bubble” with both antigen and PCR negative test, showed a positive PCR test at one of the protocol controls. In both cases the whole Paralympic team was immediately placed in quarantine.

All cases went back to the following retreats with negative PCR test and entered the bubble with a negative PCR test.

One of the two para-athletes had previously symptomatic COVID-19 (fever and fatigue) with a positive swab confirmed in mid-December. At the time there was not on-going retreats because of the Christmas holiday period. By the end of December, the athlete had another swab done with a PCR negative result and therefore was allowed to come to the retreat in January 2021. In fact, the athlete resulted PCR negative at the entrance in the retreat and PCR positive at the end of the training period. The transient PCR positive result was interpreted as lagging positivity from previous infections.

The second athlete had a very similar story. He resulted positive to a PCR test in mid-May, and he was placed in quarantine for 15 days. At the end of the quarantine period, he had PCR negative test. He was then invited to participate to the Paralympic retreat and arrived at the retreat where he was tested positive to PCR. After the positive result he was sent home in quarantine while the competition was taking place in his hometown. A PCR test was conducted again the day afterwards outside the protocol by his local health center and resulted negative. With this negative PCR test, he was allowed to participate in the Qualifying Races.

In both cases there were no COVID-19 outbreaks related to the identification of these PCR positive tested para-athletes.

## Discussion

The protocol has confirmed that it is possible to develop an anti-COVID-19 protection “bubble” where athletes and technical staff can train and also compete in safe conditions. In particular, the study indicated that it is feasible to implement a rigid prevention protocol based on repeated COVID-19 antigenic and molecular tests before, during and at the end of retreat periods and competitions.

As requested by the feasibility criteria, all personnel included in the study were highly compliant with the roles and regulations requested by the protocol and 100% of the considered study members participated in the protocol for the whole study period (10 months).

For 10 months the oropharyngeal sample collection was done always, except for two retreats, by the same member of the para-rowing team. This strategy has allowed the implementation of the “bubble” both for Paralympic retreats and competition settings using a standardized operator through a standardized procedure.

The laboratories COVID19 Center in Rome and the Laboratory at the Milan University were always able to provide fast molecular test results at the time required to allow the team to enter and to exit the “bubble.” This may seem an irrelevant detail, however the pandemic conditions required overwhelmed laboratories to perform high quality extra-work in short-well-defined time intervals.

During the 10 month-protocol implementation there were no COVID-19 outbreaks among the para-athletes and technical personnel included in the “bubble.” However, we should not derive any conclusion about the efficacy of the “bubble” to prevent COVID-19 outbreak. The protocol was implemented without a control group because pandemic conditions made it difficult to carry out a randomized and control trial for essentially ethical reasons. In full pandemic period, it was difficult to justify the creation of a protective “bubble” where some para-athletes could enter, and others could not. Furthermore, the limited number of members of the Paralympic Italian Rowing Federation Team would have made a comparison study equally inefficient. We can though describe, through a real-life example, how professional athletes are at risk of contagion and sources of outbreaks themselves if solid anti-COVID-19 protection protocols are not implemented. A COVID-19 outbreak occurred at European Rowing Championships in 2020 and 3 Olympic athletes and 2 Paralympic athletes of the Italian National rowing team resulted positive to the infection after coming home. It was unclear where and when the infection occurred because the protection protocol at that time called for a PCR test to be done in the 72 hours before departure as it was recommended for all Italian Sport Federations. There were no requirements of any diagnostic tests once athletes arrived home after competing. This set of circumstances resulted in the isolation of over 89 athletes, coaches, technical and medical staff for ~10 days including the families and all people that met those athletes once they arrived (Santis, 2020). The absence of substantial strategies to protect athletes from potentially severe health consequences also means risking infection of multiple people that contact possibly infected athletes once they returned from their championships.

The COVID-19 pandemic represents an arduous challenge for the whole of humanity. The para-athletes and athletes of the national teams represent a particular subgroup of the population for which there is an important need for protection and the implementation of preventive measures. Little is known about the modalities of viral infection impact on general health of athletes as well as the effects of the infection on their physical and mental performance. In particular, the effects of intense physical exertion on susceptibility to infection and the possibility of contagion within teams and groups of athletes and para-athletes are not known.

This study provides an important service to National and International Sports Federations in providing a tool for COVID-19 prevention. The feasibility of the protocol allows its possible implementation to all para-athletes, athletes and technical staff of different National Federations and different sports other than rowing. It might also represent an important support for infection prevention in the contest of future epidemics. The use of appropriate protocol for infection protection has also the potential to enable previously inactive (disabled) people to envisage how physical activity, even in challenging times, can become a part of their daily routines, and in ways that are enjoyable, rewarding, and meaningful (Fitzgerald et al., 2020).

## Data Availability Statement

The raw data supporting the conclusions of this article will be made available by the authors, without undue reservation.

## Ethics Statement

The studies involving human participants were reviewed and approved by University of Milan Institutional Review Board. The patients/participants provided their written informed consent to participate in this study.

## Author Contributions

GM: paper writing. PM, GB, AM, AS, SS, and AF: study conceptualization and design. GM, FM, SDe, EP, DO, FC, and AM: study operation. PM, GB, MR, and GM-S: study methodology. FP, SDe, and EP: laboratory organization and test performance. GM, FM, PM, and AM: athlete recruitment and protocol implementation. All authors contributed to the article and approved the submitted version.

## Funding

The protocol implementation was entirely supported by public funds made available by the anti-COVID19 Center of the Lazio Region, Italy.

## Conflict of Interest

The authors declare that the research was conducted in the absence of any commercial or financial relationships that could be construed as a potential conflict of interest.

## Publisher's Note

All claims expressed in this article are solely those of the authors and do not necessarily represent those of their affiliated organizations, or those of the publisher, the editors and the reviewers. Any product that may be evaluated in this article, or claim that may be made by its manufacturer, is not guaranteed or endorsed by the publisher.
